# Exposure to Ionizing Radiation during Dental X-Rays Is Not Associated with Risk of Developing Meningioma: A Meta-Analysis Based on Seven Case-Control Studies

**DOI:** 10.1371/journal.pone.0113210

**Published:** 2015-02-06

**Authors:** Ping Xu, Hong Luo, Guang-Lei Huang, Xin-Hai Yin, Si-Yang Luo, Ju-Kun Song

**Affiliations:** Gui Zhou provincial people’s hospital, Guiyang 550002, PR China; Baylor College of Medicine, UNITED STATES

## Abstract

**Background:**

Many observational studies have found that exposure to dental X-rays is associated with the risk of development of meningioma. However, these findings are inconsistent. We conducted a meta-analysis to assess the relationship between exposure to dental X-rays and the risk of development of meningioma.

**Methods:**

The PubMed and EMBASE databases were searched to identify eligible studies. Summary odds ratio (OR) estimates and 95% confidence intervals (95% CIs) were used to compute the risk of meningioma development according to heterogeneity. Subgroup and sensitivity analyses were performed to further explore the potential heterogeneity. Finally, publication bias was assessed.

**Results:**

Seven case-control studies involving 6,174 patients and 19,459 controls were included in the meta-analysis. Neither exposure to dental X-rays nor performance of full-mouth panorex X-rays was associated with an increased risk of development of meningioma (overall: OR, 0.97; 95% CI, 0.70–1.32; dental X-rays: OR, 1.05; 95% CI, 0.89–1.25; panorex X-rays: OR, 1.01; 95% CI, 0.76–1.34). However, exposure to bitewing X-rays was associated with a slightly increased risk of development of meningioma (OR, 1.73; 95% CI, 1.28–2.34). Similar results were obtained in the subgroup and sensitivity analyses. Little evidence of publication bias was observed.

**Conclusion:**

Based on the currently limited data, there is no association between exposure to dental X-rays and the risk of development of meningioma. However, these results should be cautiously interpreted because of the heterogeneity among studies. Additional large, high-quality clinical trials are needed to evaluate the association between exposure to dental X-rays and the risk of development of meningioma.

## Introduction

Intracranial meningioma is a solid tumor that arises from the meninges, which protect the central nervous system. Meningioma, one of the most clinically serious tumors of the central nervous system, accounts for approximately 20% of all intracranial tumors in male patients and 38% in female patients [1–4]. The estimated prevalence of pathologically confirmed meningioma is 3.5 in 100,000 cases per year worldwide [4]. According to a report of the National Cancer Data Base, the overall 2- and 5-year survival rates for patients with meningioma are 81% and 69%, respectively [5]. Furthermore, patients with meningioma may show neurological symptoms of increased intracranial pressure (e.g., headaches, nausea, vomiting, lethargy, and papilloedema) or focal brain dysfunction (e.g., limb weakness/numbness and seizures). Pain, disability, and mortality are patient burdens that result in high costs to society. The above data highlight the importance of screening patients at highest risk and identifying potential risk factors for the development of meningioma.

Previous studies have shown that biological risk factors of ionizing radiation (IR) are associated with a high incidence of atomic bomb-induced health issues, including various types of cancer [6, 7]. Exposure to IR is considered to be strongly associated with the risk of developing meningioma. Patients who undergo routine dental examination are those most frequently affected by X-ray exposure. In 1980, Longstreth et al. first investigated the association between dental X-ray exposure and the risk of development of meningioma. They found that exposure to dental X-rays can increase the incidence of meningioma and is a strong risk factor for meningioma [8–11]. Since then, a number of observational studies have been published [12–16]. However, the findings of these studies are varied or even conflicting. Given the widespread use of dental X-rays and poor prognosis of meningioma, any risk factors for the development of meningioma would have a substantial impact on public health. Therefore, we conducted a meta-analysis of case-control studies to evaluate the association between exposure to dental X-rays and the risk of development of meningioma.

## Methods

### Search strategy

We searched the PubMed and EMBASE databases up to March 2014 to identify relevant studies that evaluated the association between exposure to dental X-ray and the risk of development of meningioma. The following search terms were employed: (1) “meningioma(s),” “brain neoplasm(s),” “brain tumor(s),” “brain Neoplasm(s),” “meningeal Neoplasm(s),” and (2) “dental x-ray(s),” “tooth radiography,” “teeth radiography,” and “dental radiography.” Furthermore, we reviewed the reference lists of all eligible articles.

### Eligibility criteria

Studies were considered eligible for inclusion in the meta-analysis if they met the following criteria: they evaluated the association between exposure to dental, full-mouth panorex, and bitewing X-ray exposure and the risk of development of meningioma; the full text of the article was available; and they provided the adjusted and/or unadjusted odds ratio (OR), relative risk (RR) with corresponding 95% confidence interval (95%CI), or raw data with which to estimate the crude OR or RR. Studies were excluded if they met the following criteria: they were letters, comments, correspondence, conference reports, or laboratory studies or they did not contain enough data with which to calculate the OR. When multiple publications covered the same study population, only the study with the larger sample was included. Two authors (J.K.S. and X.H.Y.) independently assessed the inclusion of all retrieved studies and resolved any disagreements through discussion or consultation with a third author (G.L.H.).

### Data extraction

Two authors (J.K.S. and X.H.Y.) independently collected the following basic information using a standardized data extraction form: first author’s name, year of publication, study design, source of control, study location, number of participants (cases/controls), crude and/or adjusted point estimates and corresponding 95%CIs, and covariate features included in the multivariable model. Disputes were resolved by discussion and consensus with a third author (G.L.H.).

### Quality assessment

We evaluated the methodological quality of each study using the Newcastle-Ottawa scale (NOS) [17]. Three major components were judged as follows: representativeness of the study groups (0–4 points), determination for interested exposure in the studies (0–3 points), and comparability of groups (0–2 points). A higher score indicated better methodological quality. The quality of each study was graded as either low-level (0–4 points) or high-level (5–9 points).

### Statistical analysis

We used the OR with 95%CI as a common measure across all eligible studies. Because meningioma is a relatively rare disease, differences among the estimates of relative risk were ignored and the RR was directly converted to the OR. We used the Cochrane Q test to evaluate statistical heterogeneity. A P value of <0.05 and/or an I^2^ statistic of >50% was considered statistically significant. A random-effects model was used if heterogeneity was observed, while a fixed-effects model was used if the P value was >0.05 and/or the I^2^ statistic was <50%. We further performed sensitivity analyses to evaluate robustness and stability by sequential omission of one study in each turn. Moreover, subgroup analyses were performed to explore the potential presence of heterogeneity and assess the influence of different inclusion criteria on the overall estimate. Publication bias was evaluated using the Begg and Egger tests [18, 19]. All statistical analyses were carried out using STATA version 12.0 (Stata Corporation, College Station, TX, USA).

## Results

### Study selection

**Figure 1:**
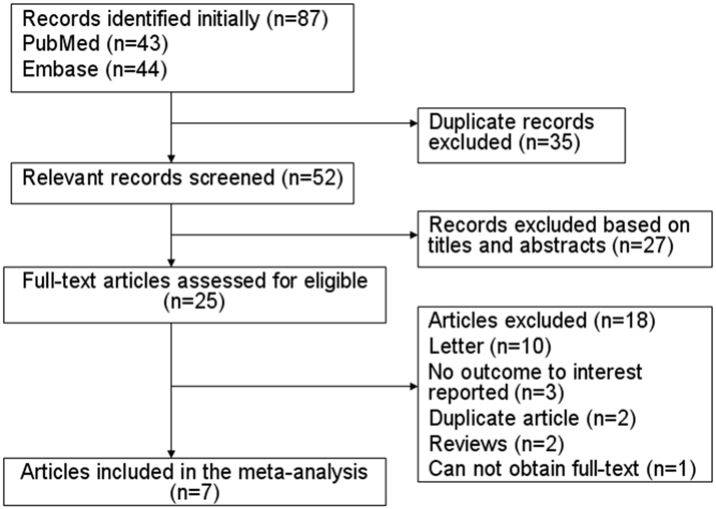
Flow chart of identification of eligible studies to final inclusion.

Fig. 1 shows a flow chart of the inclusion criteria. In total, 87 studies were screened in the initial search; 35 studies were excluded because they were duplicates, and 27 were excluded based on their titles and abstracts. Twenty-five full-text articles were reviewed for further assessment. Eighteen of these 25 articles were subsequently excluded because they were letters [20–29], had no outcome of interest [30–32], were literature reviews [3, 33], were duplicate studies [10, 11], or had no full text available [34]. Finally, seven studies were considered eligible for inclusion in the meta-analysis (Fig. 1).

### Study characteristics

The characteristics of all included articles are presented in Table 1. Seven case-control studies including 19,459 individuals and 6,174 incident cases were identified. These studies were published from 1980 to 2013. Among all studies, the sample size ranged from 342 to 20,615. Four studies were conducted in the United States [8, 9, 13, 16], one in Sweden [14], one in Taiwan [12], and one in Australia [15].

**Table 1:** Characteristics of studies included in the meta-analysis.

**Study**	**Year**	**Country**	**Study design**	**No. of subjects**	**No. of patients**	**Sex**	**Age, median (range), yrs**	**Exposure**	**OR/RR** **(95%CI)**	**Adjustment for covariates**
Preston-Martin S	1980	United States	PCC	185	189	F	18–64	Full-mouth dental X-rays	1.2 (0.58–2.49)	NA
Preston-Martin S	1989	United States	PCC	272	70	M	25–69	Dental X-rays	1.21 (0.66–2.24)	NA
								Full-mouth X-rays	2.05 (0.87–4.85)	
Ryan P	1992	Australia	PCC	417	60	F/M	NA	Dental diagnostic X-rays	0.42 (0.24–0.76)	Adjusted for age and sex
Rodvall Y	1998	Sweden	PCC	343	99	F/M	25–74	Dental X-rays	2.1 (0.41–3.52)	NA
Longstreth WT Jr	2004	United States	PCC	400	200	F/M	18 to >70	Posterior bitewings X-rays	1.25 (0.72–2.15)	Adjusted for age, sex, and education
								Full-mouth series X-rays	1.28 (0.56–2.92)	
								Panoramic X-rays	0.84 (0.51–1.40)	
								Lateral cephalometric X-rays	0.88 (0.60–1.29)	
Claus EB	2012	United States	PCC	1350	1433	F/M	20–79	Dental X-ray exposure dental X-rays	0.8 (0.6–0.9)	Adjusted for age, sex, race, education (≤16 years versus >16 years) and history of head CT
								Full-mouth X-rays	1.0 (0.9–1.3)	
								Bitewings X-rays	2.0 (1.4–2.9)	
								Panorex X-rays	1.1 (0.8–1.6)	
Lin MC	2013	Taiwan	PCC	16492	4123	F/M	44.2	Dental diagnostic X-rays	1.39 (1.30–1.50)	Adjusted for age, sex, dementia, and epilepsy

NA, not available; M, male; F, female; PCC, population-based case-control

All seven studies reported exposure to dental X-rays; four reported exposure to full-mouth series [8, 9, 13, 16], one reported exposure to bitewing X-rays, and one reported exposure to panorex X-rays [13, 16]. Five studies evaluated the OR of developing meningioma [8, 9, 12, 13, 16], and two evaluated the RR of developing meningioma [14, 15]. One study investigated only men [8], one investigated only women [9], and five investigated both men and women [12–16]. The association between exposure to dental X-rays and the risk of development of meningioma was the primary outcome in two studies [13, 16], while it was a secondary outcome in five studies [8, 9, 12, 14, 15]. Three studies did not adjust for confounding factors [8, 9, 14], whereas the others controlled for various risk factors for meningioma including age, sex, education, and others [12, 13, 15, 16].

We used the NOS to evaluate the quality of the included studies (Table 2). The median NOS score of the eligible studies was 5.0 (range, 3–7).

**Table 2:** Quality assessment of included studies based on Newcastle–Ottawa scale.

**Author**	**Year**	**Selection**	**Comparability**	**Exposure**
Preston-Martin S	1980	2	1	1
Preston-Martin S	1989	2	1	1
Ryan P	1992	2	0	1
Rodvall Y	1998	2	2	1
Longstreth WT Jr	2004	2	2	2
Claus EB	2012	3	2	2
Lin MC	2013	3	1	1

### Exposure to dental X-rays and risk of developing meningioma

**Figure 2:**
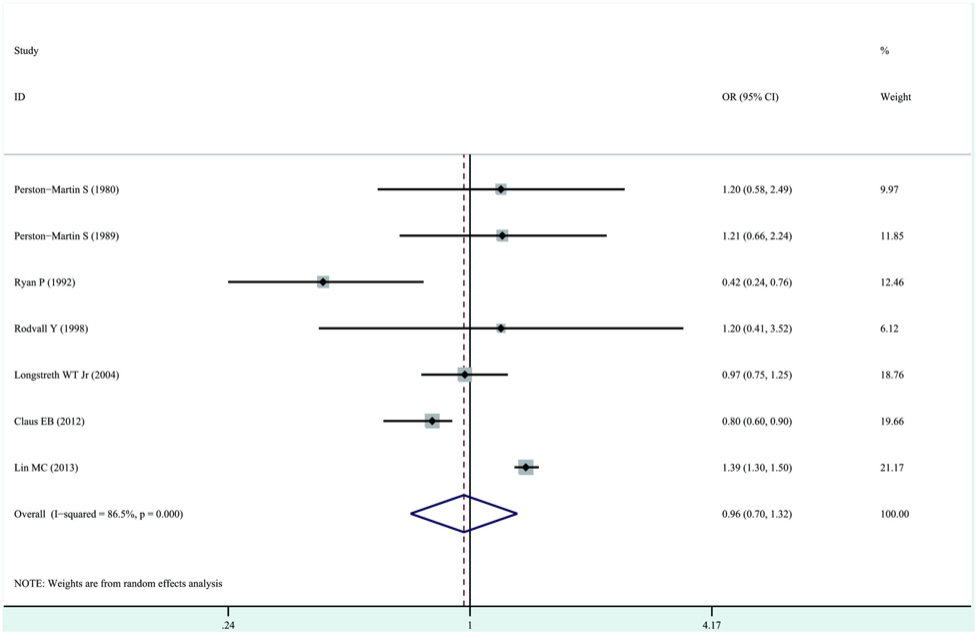
Forest plot of exposure to dental X-rays and risk of meningioma. Studies are pooled with a random-effects model.

The overall ORs were pooled to obtain the total estimate of risk using a random-effects model (OR, 0.97; 95%CI, 0.70–1.32; P = 0.82) with significant heterogeneity (P < 0.001, I^2^ = 86.5%). The results suggested that exposure to dental X-rays has no important effects on the risk of development of meningioma, and substantial heterogeneity was observed (Fig. 2). We subsequently conducted sensitivity analyses to test the stability and robustness of these results. The exclusion of any single study did not materially affect the overall combined OR, which ranged from 0.93 (95%CI, 0.66–1.32) to 1.08 (95%CI, 0.81–1.45); substantial heterogeneity was observed. When we excluded the study by Lin et al. [12], who reported an association between exposure to dental X-rays and the risk of development of benign brain tumors, we obtained analogous results (OR, 0.86; 95%CI, 0.67–1.11) with moderate heterogeneity (P = 0.086, I^2^ = 48.2%). The subgroup analyses based on different exclusion criteria yielded similar results (see Table 3).

**Table 3:** Summary of results.

	**Studies, N**	**Cases, N**	**Controls, N**	**OR (95%CI)**	**P-value**	**P of heterogeneity**	**I^2^ (%)**
Total	7	6,174	19,459	0.97 (0.70–1.32)	0.822	0.000	86.5
**Country**							
United States	4	1,892	2,207	0.90 (0.77–1.05)	0.169	0.374	3.7
Austria	1	60	417	0.42 (0.24–0.76)	0.003	NA	NA
Sweden	1	99	343	1.20 (0.41–3.52)	0.740	NA	NA
China	1	4,123	16,492	1.39 (1.30–1.50)	0.000	NA	NA
**Size effect**							
OR	5	6,015	18,699	1.08 (0.80–1.47)	0.643	0.000	86.8
RR	2	159	760	0.64 (0.23–1.76)	0.388	0.092	64.9
**Sample size**							
Large	4	5,945	18,427	1.06 (0.74–1.49)	0.755	0.000	90.1
Small	3	229	1,032	0.81 (0.37–1.76)	0.590	0.0310	71.3
**Adjustment for covariates**							
Yes	4	5,816	18,659	0.88 (0.59–1.31)	0.522	1.000	0.0
NA	3	358	800	1.21 (0.78–1.85)	0.395	0.000	93.3
**NOS score**							
High	4	5,855	18,585	1.05 (0.73–1.50)	0.621	0.021	74.2
Low	3	319	874	0.83 (0.40–1.72)	0.795	0.000	90.1

OR, odds ratio; CI, confidence interval; NA, not available; Large, ≥100 cases; Small, <100 cases; High, NOS score of ≥5; Low, NOS score of <5

### Exposure to full-mouth X-rays and risk of developing meningioma

**Figure 3:**
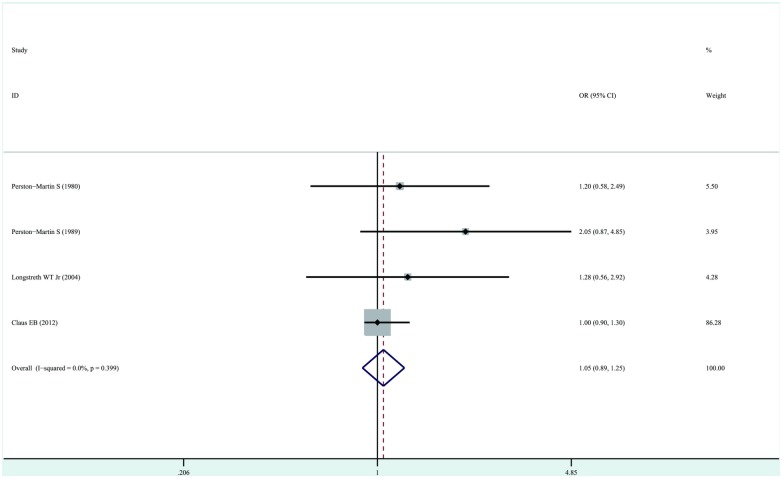
Forest plot of exposure to dental full-mouth X-rays and risk of meningioma. Studies are pooled with a fixed -effects model.

Four studies examined the association between exposure to full-mouth dental X-rays and the risk of development of meningioma [8, 9, 13, 16]. The overall OR estimates for each study were pooled to obtain the total estimate of risk using a fixed-effects model (OR, 1.05; 95%CI, 0.89–1.25; P = 0.57), and the test for heterogeneity revealed no statistical significance (P = 0.40, I^2^ = 0.0%). These results suggested that exposure to dental X-rays has no important effects on the risk of development of meningioma. No significant heterogeneity was observed (Fig. 3).

### Exposure to dental bitewing X-rays and risk of developing meningioma

**Figure 4:**
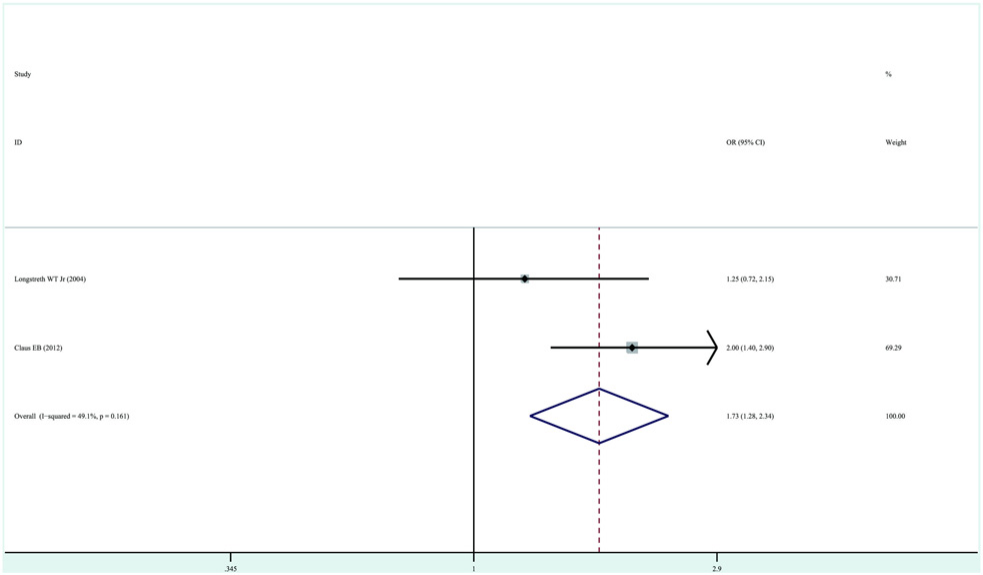
Forest plot of exposure to dental bitewing X-rays and risk of meningioma. Studies are pooled with a fixed -effects model.

Two studies contained data on exposure to dental bitewings X-rays [13, 16]. The overall OR estimates for each study were pooled to obtain the total estimate of risk using a fixed-effects model (OR, 1.73; 95%CI, 1.28–2.34; P = 0.00) with low heterogeneity (P = 0.16, I^2^ = 49.1%). These results suggest that exposure to dental bitewing X-rays is associated with a slightly increased risk of development of meningioma. Substantial heterogeneity was observed (Fig. 4). However, because only two studies reported the association, these results should be interpreted with caution.

### Exposure to dental panorex X-rays and risk of developing meningioma

**Figure 5:**
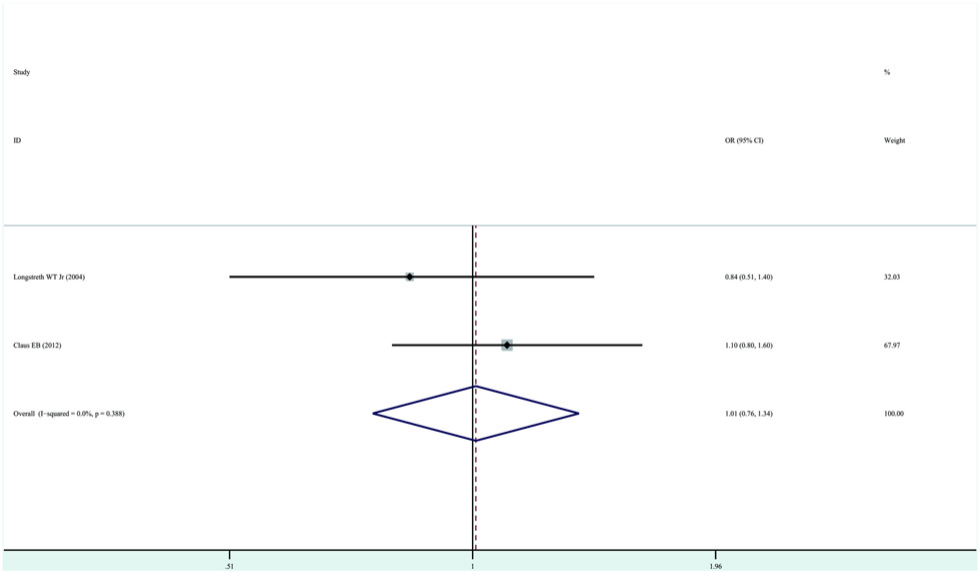
Forest plot of exposure to dental panorex X-rays and risk of meningioma. Studies are pooled with a fixed-effects model.

A pooled analysis of two studies [13, 16] showed that exposure to dental panorex X-rays does not increase the risk of development of meningioma (OR, 1.01; 95%CI, 0.76–1.34; P = 0.95). No significant heterogeneity was detected (P = 0.39, I^2^ = 0.0%) (Fig. 5).

### Publication bias

**Figure 6:**
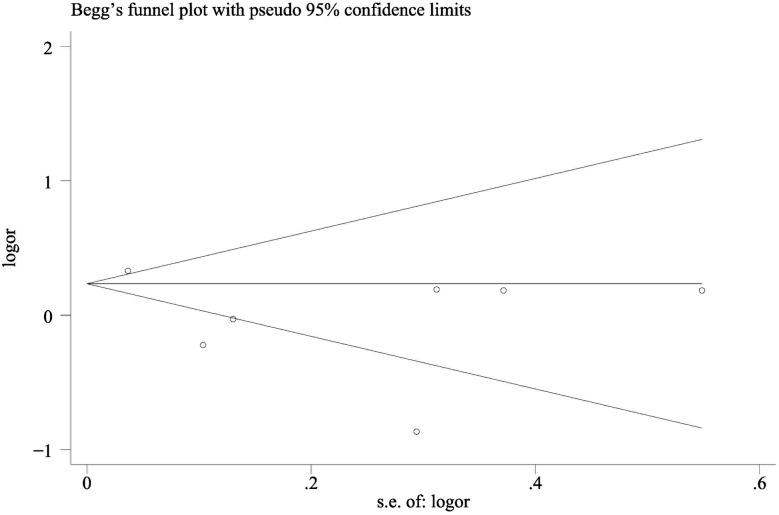
Funnel plots of exposure to dental X-rays and risk of meningioma for assessment of publication bias.

Both the Begg rank correlation test and the Egger funnel plot asymmetry test (regression method) in the meta-analysis indicated no significant publication bias (exposure to dental X-rays: Begg test, P = 0.37; Egger test, P = 0.14) (Fig. 6). Because of the limited number of articles, we did not assess the publication bias for the other outcomes.

## Discussion

The pathogenetic mechanism of meningioma remains unknown. Many conditions have been identified as risk factors, including brain injury, smoking, chronic virus infection, and occupational exposure. The most threatening risk factor for the development of meningioma is exposure to IR. Notably, a moderate to high dose of IR could increase the risk of developing various cancers as confirmed by studies of atomic bomb survivors and children irradiated for benign medical conditions and primary tumors. However, most of the general population receives lower-dose exposure during procedures such as diagnostic radiography, computed tomography (CT), or other types of radiation. The precise nature of the relationship between exposure to IR (especially low-dose IR) and the development of meningioma is not well characterized. With the progression of medical technology, medical diagnostic X-ray instruments have become widely used among the general population. Dental X-rays have been widely employed since 1919 [35]. Four radiographic techniques are commonly applied during dental examinations: periapical films, panoramic films, lateral skull or cephalometric (temporomandibular joint) films, and dental CT. The dose to which the patient is exposed is 0.018 to 1.200 mSv for periapical films, 0.135 to 0.900 mSv for panoramic films, and 0.030 to 0.200 mSv for diagnostic X-ray films [16, 36]. Although dental X-ray equipment emit a very low dose of X-rays to which the patient is exposed, there is a long-standing dispute about the association between exposure to dental X-rays and the risk of development of meningioma. Several observational studies have evaluated the association between the risk of development of meningioma and exposure to lower doses of X-rays among the general population; however, the findings are limited due to the relatively small sample sizes [8–10, 14, 15]. In their reviews of rodent studies, Claus et al. [16] and Lin et al. [12] reported that exposure to some dental X-rays appears to be associated with an increased risk of intracranial meningioma. However, some discrepancies were noted in the evaluated studies [20–29]. Therefore, a meta-analysis is required to merge and assess these findings.

The meta-analysis design serves as a valuable tool with which to study the rare effects of an intervention or treatment, permitting data synthesis and providing more convincing estimates of effect. To the best of our knowledge, this is the first meta-analysis to explore the role of exposure to dental X-rays in patients with meningioma. The overall results of the present meta-analysis of seven case-control studies using a random-effects model provide evidence that exposure to dental X-rays is not likely to have any important effects on the risk of development of meningioma. The pooled estimates were robust across the sensitivity analyses, and no publication bias was observed. Exposure to dental full-mouth and panorex X-rays may not increase the risk of development of meningioma, while exposure to dental bitewing X-rays may slightly increase the risk. These findings can partly explained as follows. First, only two studies reported an association between exposure to dental bitewing X-rays and the risk of development of meningioma. Second, patients who undergo dental bitewing examination are exposed to a relatively higher dose of X-rays. However, because of the few studies that reported this association, the present meta-analysis does not have enough power for a decisive conclusion. Further studies should focus on this association. Only three studies reported the frequency of dental X-rays, and all showed negative associations. Because the classification of exposure differed among the studies, it is difficult to merge the results using a meta-analysis.

Some limitations should be considered in the present meta-analysis. First, this meta-analysis was based on case-control studies. Although the case-control study is the most appropriate design for exposure causing rare event, this design has inherent limitations such as selective bias and recall or memory bias. Additionally, some confounding factors (e.g., race, sex, head trauma, history of head CT) are difficult to control in case-control studies. Second, substantial heterogeneity was a potential problem when interpreting the results of our analysis. This heterogeneity was not unexpected considering the differences in the characteristics of the study designs, populations sources, methods of information collection, and methods of ascertainment of exposure and outcomes among the included studies. After revealing the substantial heterogeneity, we performed subgroup analyses and obtained similar findings. Furthermore, sensitivity analyses were conducted and yielded similar results. When the study conducted by Lin et al. [12] was excluded, similar results were obtained with moderate heterogeneity. This may be because their study mainly described patients with benign brain tumors, which differs from other studies. However, pooled effect estimates based on heterogeneous data should be interpreted with caution. Third, the statistical strength for bitewing and full-mouth panorex X-ray examinations was limited because of the relatively small number of eligible studies. Finally, only four studies controlled for confounders; this may have more accurately reflected the association between exposure to X-rays and the risk of development of meningioma than the use of unadjusted ORs. However, in the subgroup analysis, similar results were observed between studies with adjusted and unadjusted ORs. Overall, these limitations may have affected our final results.

The following points should be considered for further studies. First, it is necessary to establish a standardized protocol with respect to exposure dose, method of examination, and duration of exposure to dental X-rays because great variation exists in the literature. Second, more large-scale studies should be performed on the relationship between various types of dental X-ray exposure and the risk of development of meningioma. Finally, the average follow-up period was not reported in this meta-analysis; longer-term studies are needed.

In conclusion, the currently available evidence indicates that exposure to dental X-rays is unlikely to have any important effects on the risk of development of meningioma. Although these findings are encouraging, the results of this meta-analysis should be interpret with caution because of the heterogeneity among the studies and the relatively limited number of studies, Further large-scale, well-designed trials on this topic are needed.

## Supporting Information

S1 PRISMA Checklist (DOC)
